# Impact of Re-Warm-Up During Resistance Training: Analysis of Mechanical and Physiological Variables

**DOI:** 10.3390/sports13050142

**Published:** 2025-05-07

**Authors:** Pedro P. Neves, Diogo L. Marques, Henrique P. Neiva, Daniel A. Marinho, Ricardo Ferraz, Mário C. Marques, Ana R. Alves

**Affiliations:** 1 Department of Sport Sciences, University of Beira Interior, 6201-001 Covilhã, Portugal; pedro.miguel.neves@ubi.pt (P.P.N.); diogo.marques@ubi.pt (D.L.M.); hpn@ubi.pt (H.P.N.); dmarinho@ubi.pt (D.A.M.); ricardo.ferraz@ubi.pt (R.F.); mariomarques@mariomarques.com (M.C.M.); 2 Research Center in Sports Sciences, Health Sciences and Human Development (CIDESD), Contento de Santo António, 6201-001 Covilhã, Portugal

**Keywords:** pre-exercise, specific warm-up, strength training, propulsive velocity, power output, heart rate, lactate, rate of perceived exertion, exercise sequence, young adults

## Abstract

**Objectives:** This study examined the effects of re-warm-up versus no re-warm-up before squats or bench presses on mechanical, physiological, and psychophysiological responses in recreationally trained men. **Methods:** Twenty-two participants (22.8 ± 3.3 years) completed four randomized sessions involving different re-warm-up and exercise sequences. Measurements included heart rate, blood lactate, tympanic temperature, and perceived exertion. Key performance metrics, such as mean propulsive velocity, peak velocity, power, velocity loss, and effort index, were analyzed. **Results:** Findings revealed that re-warming up before squats (W + BP + RW + SQ) significantly enhanced propulsive velocity and power compared to no re-warm-up (W + BP + SQ) (*p* ≤ 0.05; *d* = 0.45–0.62). However, re-warming up before the bench press (W + SQ + RW + BP) did not improve mechanical performance compared to the standard sequence (W + SQ + BP) (*p* > 0.05; *d* = 0.10–0.38). Notably, velocity loss and effort index were higher in the third bench press set under the W + SQ + BP condition (*p* ≤ 0.05; *d* = 0.53–0.60). No significant differences in physiological or psychophysiological responses were found between conditions. **Conclusions:** Overall, re-warm-up effectively improved squat mechanical performance when performed after the bench press but had a minimal impact on the bench press when performed after squats. These findings suggest that re-warming up before lower-body exercises may enhance mechanical performance, while its benefits may be less pronounced for upper-body exercises.

## 1. Introduction

Warm-up exercises are crucial in recreational training and competition as they prepare the body physiologically and psychologically, reduce injury risk, and enhance performance across individual and team sports [1,2,3,4]. Different warm-up strategies have been investigated, and research tends to agree regarding their positive impact on physical performance [5,6,7,8]. For instance, warm-up by running or cycling for 15 min at 80% of maximum oxygen uptake improves vertical jump performance immediately and in the following 20 min post-warm-up [9,10]. Furthermore, warm-up by running 500 m at 70% running intensity followed by 3 × 250 m at 100% running intensity significantly improves 5000 m running performance in trained endurance runners [11].

In resistance training, extensive research has been conducted to analyze the impact of post-activation potentiation (PAP), the role of general and/or specific warm-up procedures, and the effects of various warm-up protocols (e.g., varying volumes and intensities) [12,13,14,15]. Studies have documented the PAP effect following various exercises, including maximal loaded, submaximal loaded, and unloaded exercises [16,17,18]. According to Wilson et al. [18], the potentiation of warm-up can be optimized with multiple sets performed at moderate intensities (i.e., 60–84% of 1RM) and rest periods between 7 and 10 min in practitioners with at least one year of resistance training background. Furthermore, combining general and specific warm-ups improves force production and increases strength in the one-repetition maximum (1RM) test compared to just performing a specific warm-up during 1RM testing procedures [19]. A study by Barnes et al. [2] demonstrated that a specific warm-up tailored to the exercise led to a more significant enhancement in peak power for the high pull than a general warm-up.

While many previous studies have shown the benefits of warming up before resistance training, few have explored different strategies, including monitoring movement velocity, to quantify training intensity and the level of effort following specific warm-up strategies [20,21,22]. Measuring movement velocity in real time in a resistance training setting offers a reliable and regular means of monitoring exercise intensity and effort [23,24,25]. The measurement of repetition velocity is an accurate and objective indication of the actual exertion and level of effort experienced by the practitioner during training, providing valuable information to strength and conditioning coaches [23,25]. Following this velocity-monitored resistance training approach, recent findings have demonstrated that specific warm-ups comprising two sets of six repetitions performed with maximal intended velocities at 40% and 80% of the training load (i.e., 32 and 64% of 1RM) enhance neuromuscular function, enabling higher movement velocity outputs in the initial squat and bench press repetitions and achieving peak velocities more quickly [22]. Furthermore, a specific warm-up of one set of six repetitions performed with maximal intended velocities at 80% of 1RM seems more effective in potentiating mechanical performance in the squat than at 40% of 1RM in resistance-trained males [21]. On the other hand, a specific warm-up involving two sets of six repetitions performed with maximal intended velocities with progressive loads (40% to 80% of 1RM) may be more effective in increasing mechanical performance in the bench press than a single set [21].

Although efforts have been made to understand the effect of warming up in its various forms (e.g., general and specific) on force production and strength performance [19,26,27], resistance training is typically not just a single exercise but a sequence of different exercises targeting the same or different muscle groups [28]. In this respect, it is particularly important to understand whether re-warming before subsequent strength exercises benefits mechanical (i.e., velocity) and physiological performance (e.g., heart rate and lactate responses). However, little is known about the need to re-warm-up during the session using specific warm-ups to enhance subsequent exercise performance. This need is even more evident when the muscle groups that are most stimulated differ between successive exercises. Therefore, considering that the warm-up effect may decrease throughout the session, especially during inactivity [29] and if the intensity is low [9,10], an effective and practical solution to avoid this decline becomes necessary. It is important to balance the free time available in sessions and improve physical performance through re-warming strategies between exercises, primarily when focusing on different muscle groups during sessions.

Therefore, given the research gap regarding the impact of re-warming up before subsequent strength exercises, this study aimed to analyze the effects of different re-warm-up strategies before the squat or bench press on mechanical, physiological, and psychophysiological responses in recreationally trained men. It was hypothesized that performing a specific re-warm-up following the first strength exercise of the session would improve mechanical performance in the squat and bench press and produce a similar physiological and psychophysiological response in recreationally trained men.

## 2. Materials and Methods

### 2.1. Participants

We conducted an a priori analysis in G*Power (v3.1.9.2, Dusseldorf, Germany) to determine the required sample size [30]. Considering a Cohen’s *d* of 0.72 (effect size based on an experimental study that reported a large difference in mean propulsive velocity [MPV] between experimental conditions with versus without re-warm-up [22]), an alpha level of 0.05, a statistical power of 80%, and a drop-out rate of 20%, an estimated sample size of twenty-two participants was required. Based on this estimation, we recruited twenty-two male sport sciences students aged between 19 and 32 (22.8 ± 3.3 years, 76.1 ± 12.6 kg, and 1.78 ± 0.06 m; 1RM bench press: 78.5 ± 11.6 kg; 1RM squat: 96.0 ± 23.1 kg) to participate in this study. Inclusion criteria comprised male participants aged 18 or over, without physical limitations or restrictions to perform resistance exercises, and at least 6 months of resistance training experience, especially in the bench press and squat using the Smith machine. Exclusion criteria encompassed female participants, musculoskeletal injuries in the previous three months, and no resistance training experience. Participants who met the criteria and voluntarily agreed to participate in the study were included. Each participant reported no previous illness, injury, or other physical problems that could impair their performance during resistance training sessions. All participants were verbally informed about the study procedures and signed a consent form. All procedures followed the recommendations of the Declaration of Helsinki and were approved by the Ethics Committee of the University of Beira Interior (approval number: CE-UBI-Pj-2021–018).

### 2.2. Experimental Design

**Figure 1:**
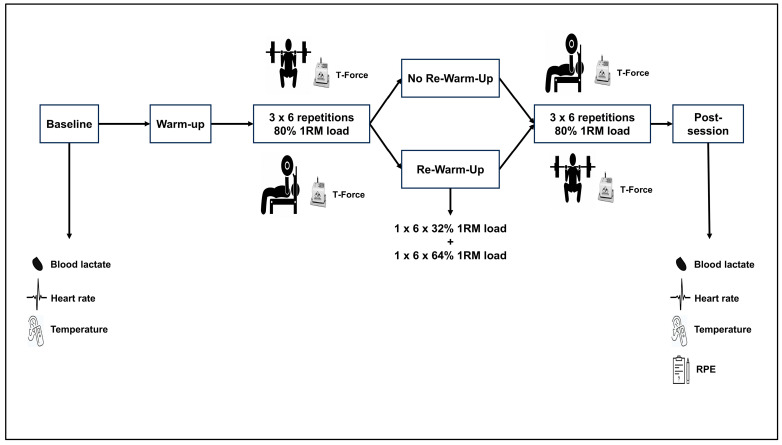
Experimental procedures and timeline of data collection. Sets × Repetitions × % 1RM load.

In a crossover design, participants performed four resistance training sessions with or without re-warm-up conditions following the first strength exercise in a randomized order with at least 48 h of rest between each. The experimental conditions were as follows: (i) Warm-Up + Squat + Bench Press (W + SQ + BP); (ii) Warm-Up + Squat + Re-Warm-Up + Bench Press (W + SQ + RW + BP); (iii) Warm-Up + Bench Press + Squat (W + BP + SQ); (iv) Warm-Up + Bench Press + Re-Warm-Up + Squat (W + BP + RW + SQ). Heart rate, blood lactate, and tympanic temperature (physiological variables) were measured once at baseline and immediately after the last exercise of the session. The rate of perceived exertion (RPE; psychophysiological variable) was also collected immediately after the last exercise of the session. In all sessions, participants performed three sets of six repetitions at 80% of 1RM in the squat and bench press. A linear velocity transducer (T-Force Dynamic Measurement System, Ergotech, Murcia, Spain) with the cable connected to the barbell of a Multipower (Multipower Fitness Line, Perola, Murcia, Spain) collected all mechanical variables during the execution of repetitions. Mechanical variables included MPV, peak velocity (PV), time to peak velocity (TPV), velocity loss (VL), mean propulsive power (MPP), peak power (PP), and bar displacement. The degree of fatigue was expressed as the effort index (EI) [31]. The experimental procedures of this study lasted three weeks, with two sessions performed per week. The first was to familiarize the participants with the testing protocols and measure height and body mass (Seca Instruments, Ltd., Hamburg, Germany). Then, participants performed a progressive loading test in the second session to determine the 1RM load in the bench press and squat. In the following sessions, participants performed the experimental conditions. After physiological baseline measurements, participants warmed up and performed three sets of six repetitions at 80% of 1RM on the Smith machine bench press or Smith machine squat, with a three-minute break period between sets. Following the first strength exercise, participants either performed a re-warm-up or the second strength exercise of the session. The re-warm-up consisted of two sets of six repetitions at 32% and 64% of 1RM on the Smith machine squat or Smith machine bench press, with a one-minute inter-set rest interval [20,21]. The bench press and squat exercises were chosen because they are widely used in strength and conditioning settings and recognized for their effectiveness in increasing muscle mass and strength in the general and athletic populations [32]. Additionally, using a Smith machine for these exercises aids in stabilizing the execution technique and ensures that the movement of the barbell and the transducer’s cable is linear, thus minimizing errors and enhancing data collection accuracy [33,34]. All sessions were supervised by the first researcher and two experienced strength and conditioning coaches. Figure 1 illustrates the experimental design.

### 2.3. Progressive Loading Test in the Bench Press and Squat

In the bench press test, participants lay supine on a flat bench with their feet on the floor and hands placed slightly wider than shoulder-width on the barbell [35,36]. They lowered the barbell to the chest, just above the nipples, in a controlled manner, and after approximately one second of pause, they performed the concentric phase as fast as possible [35]. Participants were not allowed to bounce the barbell off the chest or to raise the shoulders or trunk off the bench [37]. In the squat, participants started from an upright position with knees and hips fully extended, hands placed slightly wider than shoulder-width on the barbell, with the barbell resting on the back at the level of the acromion [38]. Then, they began to descend until the tops of their thighs were below 90º in continuous movement (eccentric phase), and immediately after, they ascended at maximum velocity to the initial position (concentric phase) [38]. Two experienced strength coaches were on both sides of the barbell to ensure safety. In both exercises, the first researcher and strength coaches controlled the movement to guarantee that all repetitions were performed with the required technique and a similar range of movement. The initial load was fixed at 17 kg and 20 kg for all participants in the bench press and squat, respectively, and gradually increased by 10 kg. The test finished when participants reached a concentric MPV of 0.40 m·s^−1^ in the bench press and 0.60 m·s^−1^ in the squat, corresponding to 85% 1RM in both exercises [25,38]. Inter-set recoveries ranged from 3 min (light loads) to 5 min (heavy loads). The 1RM load was determined from the last MPV obtained during the test as follows: (100 × Load)/(8.4326 × MPV^2^ − 73.501 × MPV + 112.33) for the bench press [25] and (100 × load)/(−5.961 × MPV^2^ − 50.71 × MPV + 117) for the squat [38].

### 2.4. Resistance Training Protocols

The warm-up included 10 min of treadmill running, starting at 50–55% of heart rate reserve until reaching 70% in the last 2 min, followed by two sets of six repetitions at 32% and 64% of 1RM in the squat or bench press (1 min inter-set rest interval) [20,21]. After the warm-up, participants performed three sets of six repetitions at 80% of 1RM in the Smith machine bench press or Smith machine squat, with a three-minute interval between sets. Then, depending on the experimental condition, participants either performed a re-warm-up of two sets of six repetitions at 32% and 64% of 1RM in the squat or bench press (1 min inter-set rest interval) or immediately performed the second exercise without re-warm-up. All concentric repetitions were performed with the maximal intended velocity, while the eccentric phase was controlled (~3 s). The first researcher and two strength coaches (each coach on each side of the barbell to spot participants) supervised all sessions to guarantee a correct execution technique and encourage participants to exert maximum effort during all repetitions.

### 2.5. Measurement of Physiological and Psychophysiological Parameters

Heart rate was monitored during all sessions with a Polar watch (Polar Vantage NV, Kempele, Finland). Blood lactate concentration was measured using a hand-held portable device (Lactate Pro 2 LT-1730, Arkray Inc., Tokyo, Japan). After cleansing the site with 70% alcohol, the fingertip was punctured using a disposable lancet. The first drop of blood was discarded, and a tiny blood sample was collected for analysis [39]. Tympanic temperature was measured with an infrared thermometer to estimate the central body temperature (Braun Thermoscan IRT 4520, Kronberg, Germany) [40]. RPE values were measured using a 15-grade scale (Borg scale 6–20) [41].

### 2.6. Statistical Analysis

Microsoft Office Excel (Microsoft Inc., Redmond, WA, USA) was used to collect physiological and psychophysiological results and extract data from the T-Force software (v. 2.35, Ergotech, Murcia, Spain). Afterward, data were analyzed in SPSS (v27.0, IBM Corp., Armonk, NY, USA). Descriptive data are presented as mean ± standard deviation and 95% confidence intervals (CIs). The Shapiro–Wilk test analyzed the normality of the data. After confirming the assumption of data normality, paired *t*-tests were used to compare experimental conditions with vs. without re-warm-up (W + SQ + BP vs. W + SQ + RW + BP; W + BP + SQ vs. W + BP + RW + SQ) in mechanical, physiological, and psychophysiological variables. The significance level was set at *p* ≤ 0.05. The effect size was calculated using Cohen’s *d* to determine the magnitude of the differences between conditions. The effect size was interpreted as trivial (*d* = 0.00–0.19), small (*d* = 0.20–0.59), moderate (*d* = 0.60–1.19), large (*d* = 1.20–1.99), very large (*d* = 2.00–3.99), and extremely large (*d* > 4.00) [42]. Figures were generated in GraphPad Prism (v7.0, GraphPad Inc., San Diego, CA, USA).

## 3. Results

### 3.1. Mechanical Differences Between Experimental Conditions with vs. Without Re-Warm-Up

Table 1 shows significant differences between W + BP + SQ vs. W + BP + RW + SQ conditions on MPV (first set: t_21_ = −2.43, *p* = 0.02, *d* = 0.52; second set: t_21_ = −2.25, *p* = 0.04, *d* = 0.48; third set: t_21_ = −2.09, *p* = 0.05, *d* = 0.45), PV (first set: t^21^ = −2.89, *p* = 0.01, *d* = 0.62; second set: t_21_ = −2.69, *p* = 0.01, *d* = 0.57), MPP (second set: t_21_ = −2.57, *p* = 0.02, *d* = 0.55; third set: t_21_ = −2.10, *p* = 0.05, *d* = 0.45), and PP (second set: t_21_ = −3.11, *p* = 0.01, *d* = 0.66; third set: t_21_ = −2.18, *p* = 0.04, *d* = 0.47).

Table 2 shows significant differences between W + SQ + BP vs. W + SQ + RW + BP on VL (third set: t_21_ = 2.48, *p* = 0.02, *d* = 0.53), TPV (second set: t_21_ = 2.98, *p* = 0.01, *d* = 0.64), and EI (third set: t_21_ = −0.23, *p* = 0.01, *d* = 0.60).

### 3.2. Physiological and Psychophysiological Differences Between Experimental Conditions with vs. Without Re-Warm-Up

**Figure 2:**
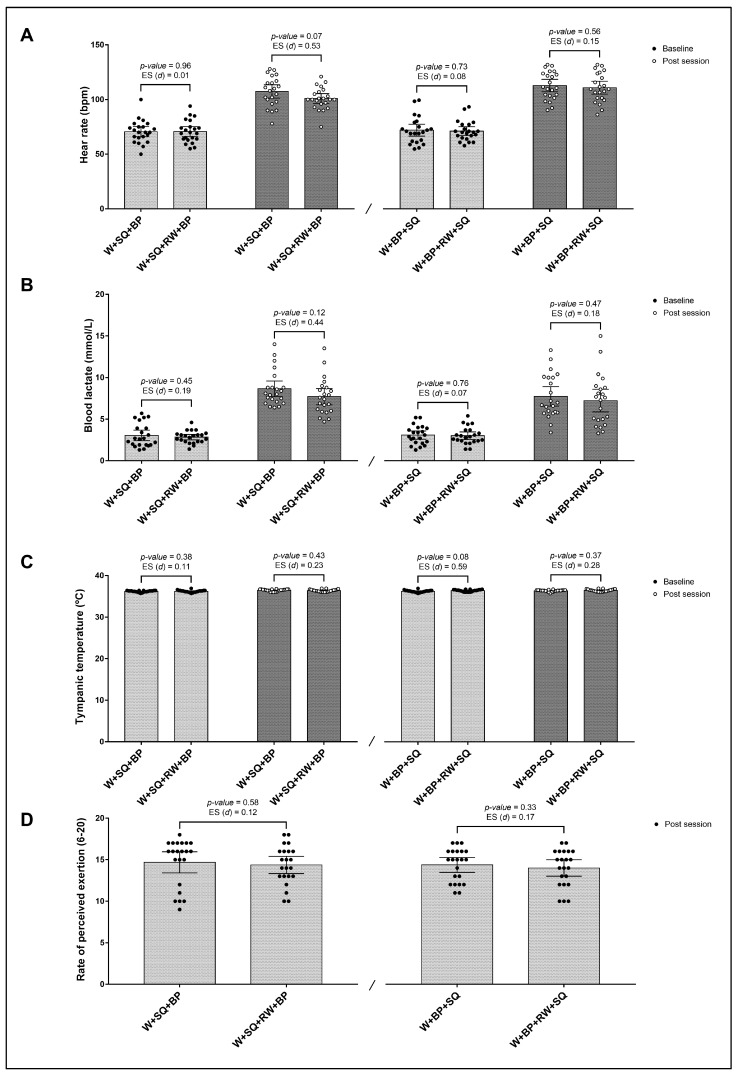
Differences between conditions with and without re-warm-up on heart rate (**A**), blood lactate (**B**), tympanic temperature (**C**), and rate of perceived exertion (**D**) at baseline and post-session. W + SQ + BP: Warm-Up + Squat + Bench Press; W + SQ + RW + BP: Warm-Up + Squat + Re-Warm-Up + Bench Press; W + BP + SQ: Warm-Up + Bench Press + Squat; W + BP + RW + SQ: Warm-Up + Bench Press + Re-Warm-Up + Squat. ES: Cohen’s *d* effect size.

Figure 2 shows no significant differences in physiological and psychophysiological parameters between W + SQ + BP vs. W + SQ + RW + BP and W + BP + SQ vs. W + BP + RW + SQ at baseline and post-session.

## 4. Discussion

This study aimed to analyze the effects of re-warm-up after the first strength exercise of the session (bench press or squat) on mechanical, physiological, and psychophysiological parameters in recreationally trained men. The hypothesis that the re-warm-up would improve mechanical performance in the squat and bench press was partially confirmed by the present findings. Re-warming up between the bench press and squat (W + BP + RW + SQ condition) was shown to be more favorable for enhancing MPV and PV during the first two sets of squats than not performing sets of re-warm-up (W + BP + SQ condition). Moreover, the power-related variables were higher in the second and third sets when the re-warm-up was performed. On the other hand, the re-warm-up between the squat and bench press (W + SQ + RW + BP condition) did not show differences in the propulsive velocity compared to the no-re-warm-up condition (W + SQ + BP condition). However, the relative VL and EI were higher in the third set of the bench press when no re-warm-up was completed after the squat. The comparison between experimental conditions with vs. without re-warm-up revealed no significant differences in physiological and psychophysiological parameters, confirming our second hypothesis that an additional warm-up in the session would not cause a disturbance in physiological parameters. These results highlight the importance of re-warming up during the resistance training session to optimize mechanical performance, primarily when the following exercise works different muscle groups compared to the previous exercise.

In our study, the re-warm-up effect optimized the mechanical performance in the squat exercise, which aligns with previous studies that observed similar mechanical responses [21,22]. This result can occur because the squat recruits large muscle groups, such as the quadriceps femoris and gluteus maximus [43], which may benefit from a pre-activation to maximize force production and propulsive velocity. Contrarily, when the re-warm-up was performed between the squat and bench press (W + SQ + RW + BP condition), it did not result in any additional effect on the mechanical performance of the bench press. This result may be explained because the bench press, contrary to the squat, involves smaller muscle groups than the squat [31,44], which may benefit from the muscular activation performed in the previous and different exercises, including the squat.

Over the years, the warm-up phase has been considered a critical component of a training session to potentiate the performance of athletes in different sports [6]. It is well known that the warm-up routine increases body temperature, preparing the body for the following activity, which can produce positive effects on physiological parameters, such as an increment in metabolic efficiency or nerve conduction rate and a decrease in muscular stiffness [45,46]. Despite the benefits of the warm-up, some uncertainties remain regarding re-warm-ups’ effects in specific resistance training activities [12,26]. Following the American College of Sports Medicine guidelines [47] for resistance training prescription to novice and untrained individuals, it is recommended to prescribe exercises focusing on full-body exercises, with a special emphasis on applying a general warm-up at the beginning of the session. However, there is still a gap in the literature about the contribution of warm-up or re-warm-up to resistance training performance.

Our results reported that re-warming up between the squat and bench press (W + SQ + RW + BP) did not appear to enhance physical performance, as the propulsive velocity did not differ from the experimental condition without re-warm-up (W + SQ + BP). However, the TPV in the bench press was inferior in the second set using the re-warm-up condition, which can be important for practitioners who aim to increase the ability to produce force quickly. Furthermore, it is relevant to highlight the lower VL and EI measured in the last set performed on the bench press when the re-warm-up was used after the squat. Considering that EI is a powerful indicator of neuromuscular fatigue [31], the current results may support the relevance of the re-warm-up in the sense that it can contribute to attenuating fatigue levels and preserving the ability to produce force for a longer time within the training session.

Although no differences were found between experimental conditions with vs. without re-warm-up in physiological responses, re-warm-up after the squat or bench press resulted in slightly, not significantly, lower blood lactate concentrations compared to conditions without re-warm-up. These results might be related to the decrease in volume and relative intensity during the re-warm-up phase, which may also enable the body to recover between exercises. Previous research has found that performing a general warm-up (10 min of treadmill running at 70% of heart rate reserve) followed by a specific warm-up (two sets of six repetitions at 32% and 64% of 1RM) and without a re-warm-up phase after the first strength exercise of the session caused a higher physiological response than just performing the specific warm-up [20]. These results reinforce the greater physiological demand of performing strength exercises continuously during the training session. A viable alternative to decrease the physiological response can be achieved by performing a re-warm-up before the following strength exercise. This strategy aims to potentiate mechanical performance during strength exercises through a progressive increase in exercise intensity (e.g., from 32% to 64% of 1RM) while allowing the simultaneous recovery of energy substrates necessary for high force production levels, such as phosphocreatine. Indeed, increasing the speed of phosphocreatine resynthesis during a short recovery phase will benefit athletes participating in activities requiring intermittent exercise, such as resistance training [48]. Therefore, if the physiological responses following the re-warm-up are slightly lower compared to no re-warm-up conditions, these practical strategies can benefit muscle force production in the subsequent strength exercises. Future studies must employ a detailed analysis using, for example, muscle temperature, hormonal responses, and motor control following resistance training strategies with and without re-warm-up for a deeper analysis of the acute physiological responses.

Some limitations of this study should be addressed. Firstly, although the sample size enabled a statistical power of 80%, including a larger sample would provide more robust conclusions and support the generalization of the effects of different resistance training warm-up strategies on mechanical, physiological, and psychophysiological parameters. Secondly, including female participants would allow us to investigate potential differences in their mechanical, physiological, and psychophysiological responses to various warm-up conditions compared to male participants. Additionally, involving elite athletes would be essential for assessing whether different physical performance levels impact the mechanical, physiological, and psychophysiological responses to various warm-up strategies. Analyzing data from men and women across different competitive levels could provide significant practical insights for prescribing resistance training, particularly regarding warm-up and re-warm-up strategies during sessions. Thirdly, only the squat and bench press were included in this study. Usually, resistance training is not constituted by only these two exercises, reinforcing the need to include more exercises in future research. Finally, incorporating more physiological variables such as creatine kinase, testosterone, and cortisol would enable a deeper analysis of how various re-warm-up strategies affect muscle damage and function biomarkers. Considering the somewhat reduced physiological responses observed after the re-warm-up techniques used in this study, could these strategies lead to lower creatine kinase and cortisol levels while increasing testosterone production? This evaluation would clarify the influence of different resistance training re-warm-up methods on muscle damage and function biomarkers in recreationally trained men. Despite this study’s limitations, the current findings provide evidence for researchers and strength and conditioning coaches regarding warm-up methodologies and their effects on resistance training performance. Future research should include additional resistance training exercises (e.g., knee extension and biceps curls) or even supplementary variables such as muscle temperature and hormonal responses to enrich the knowledge of the warm-up phenomenon in resistance training performance. These analyses could be pertinent and helpful in increasing the understanding of the effects of different warm-up routines in recreational contexts and sports performance.

This study has practical implications for researchers and strength and conditioning coaches when implementing interventions in recreationally trained males. Based on the results, when combining the bench press and squat in the same resistance training session, performing a re-warm-up following the bench press may be beneficial for improving the squat’s propulsive velocity. Furthermore, introducing a re-warm-up between the squat and bench press may help decrease the magnitude of fatigue in the final sets and preserve mechanical performance over the sets. Finally, performing a re-warm-up in either protocol will not induce a more significant increase in heart rate, blood lactate, tympanic temperature, and RPE when compared to not performing a re-warm-up.

## 5. Conclusions

The current study demonstrated that when the squat was performed after the bench press, the re-warm-up demonstrated a notable increase in propulsive velocity, indicating its positive influence on physical performance outcomes. However, when the bench press followed the squat, the impact of the re-warm-up appeared less pronounced. In terms of physiological and psychophysiological parameters, no differences were found between the experimental conditions with vs. without re-warm-up. These findings highlight the positive impact of re-warm-up between exercises on mechanical performance during resistance training, particularly in the squat, emphasizing the need for personalized approaches to optimize resistance training outcomes for recreationally trained men.

## Figures and Tables

**Table 1:** Comparison between condition with Warm-Up + Bench Press + Squat vs. condition with Warm-Up + Bench Press + Re-Warm-Up + Squat on mechanical parameters. The results correspond to the second exercise performed in the session (squat).

	W + BP + SQ	W + BP + RW + SQ	95% CI	*p*-*Value*	ES (*d*)
SET 1					
MPV (m·s^−1^)	0.58 ± 0.10	0.61 ± 0.09	[−0.05, 0.00]	**0.02**	0.52
PV (m·s^−1^)	1.10 ± 0.18	1.16 ± 0.14	[−0.10, −0.02]	**0.01**	0.62
VL (%)	17.22 ± 8.69	14.57 ± 5.48	[−0.29, 5.60]	0.08	0.40
TPV (ms)	615.14 ± 106.14	586.64 ± 97.59	[−18.78, 75.78]	0.22	0.27
EI	9.84 ± 4.44	8.93 ± 3.46	[−0.56, 2.39]	0.21	0.27
MPP (W)	431.03 ± 79.33	447.67 ± 100.16	[−43.82, 10.54]	0.22	0.27
PP (W)	957.32 ± 231.93	1006.28 ± 229.01	[−108.12, 10.20]	0.10	0.37
BD (cm)	49.04 ± 7.40	48.76 ± 7.13	[−1.88, 2.43]	0.79	0.06
SET 2					
MPV (m·s^−1^)	0.58 ± 0.10	0.60 ± 0.09	[−0.04, 0.00]	**0.04**	0.48
PV (m·s^−1^)	1.08 ± 0.16	1.13 ± 0.13	[−0.09, −0.01]	**0.01**	0.57
VL (%)	14.93 ± 6.41	15.31 ± 6.78	[−3.12, 2.36]	0.78	0.06
TPV (ms)	608.50 ± 94.25	585.36 ± 104.10	[−16.37, 62.65]	0.24	0.26
EI	8.40 ± 3.35	8.95 ± 3.45	[−2.05, 0.95]	0.46	0.16
MPP (W)	425.66 ± 88.50	445.75 ± 96.07	[−36.34, −3.84]	**0.02**	0.55
PP (W)	930.17 ± 217.11	993.68 ± 222.62	[−105.92, −21.09]	**0.01**	0.66
BD (cm)	48.55 ± 6.87	48.06 ± 7.52	[−1.16, 2.14]	0.54	0.13
SET 3					
MPV (m·s^−1^)	0.58 ± 0.11	0.60 ± 0.09	[−0.05, 0.00]	**0.05**	0.45
PV (m·s^−1^)	1.09 ± 0.18	1.13 ± 0.13	[−0.08, 0.00]	0.06	0.43
VL (%)	16.40 ± 7.81	14.64 ± 6.76	[−1.65, 5.18]	0.30	0.23
TPV (ms)	603.00 ± 116.46	568.00 ± 96.76	[−11.54, 81.54]	0.13	0.33
EI	9.31 ± 3.82	8.83 ± 4.14	[−1.56, 2.51]	0.63	0.10
MPP (W)	429.85 ± 80.85	447.75 ± 86.41	[−35.60, −0.21]	**0.05**	0.45
PP (W)	949.00 ± 242.39	995.35 ± 220.93	[−90.51, −2.17]	**0.04**	0.47
BD (cm)	48.11 ± 7.63	47.75 ± 7.33	[−1.37, 2.08]	0.67	0.09

Significant differences are marked in bold. Data are presented as means ± SD unless otherwise stated. Bold values denote significant differences. W + BP + SQ: Warm-Up + Bench Press + Squat; W + BP + RW + SQ: Warm-Up + Bench Press + Specific Re-Warm-Up + Squat; BD: bar displacement; CI: confidence interval; ES: Cohen’s *d* effect size; MPV: mean propulsive velocity; PV: peak velocity; VL: velocity loss; TPV: time to achieve peak velocity; EI: effort index; MPP: mean propulsive power; PP: peak power.

**Table 2:** Comparison between condition with Warm-Up + Squat + Bench Press vs. condition with Warm-Up + Squat + Re-Warm-Up + Bench Press on mechanical parameters. The results correspond to the second exercise performed in the session (bench press).

	W + SQ + BP	W + SQ + RW + BP	95% CI	*p*-*Value*	ES (*d*)
SET 1					
MPV (m·s^−1^)	0.46 ± 0.11	0.45 ± 0.07	[−0.04, 0.06]	0.66	0.10
PV (m·s^−1^)	0.74 ± 0.22	0.69 ± 0.12	[−0.05, 0.13]	0.35	0.21
VL (%)	24.68 ± 9.21	28.43 ± 10.41	[−10.65, 3.17]	0.27	0.24
TPV (ms)	637.45 ± 238.77	669.59 ± 208.30	[−160.57, 96.30]	0.61	0.11
EI	11.05 ± 3.93	12.66 ± 4.44	[−4.45, 1.23]	0.25	0.25
MPP (W)	276.87 ± 58.92	278.60 ± 63.77	[−27.63, 24.18]	0.89	0.03
PP (W)	475.03 ± 150.10	449.77 ± 99.62	[−39.32, 89.84]	0.43	0.17
BD (cm)	40.92 ± 4.32	40.04 ± 3.56	[−0.35, 2.10]	0.15	0.32
SET 2					
MPV (m·s^−1^)	0.46 ± 0.08	0.43 ± 0.07	[−0.01, 0.07]	0.13	0.34
PV (m·s^−1^)	0.70 ± 0.18	0.64 ± 0.11	[−0.01, 0.14]	0.09	0.38
VL (%)	29.86 ± 12.44	31.41 ± 11.70	[−8.11, 5.02]	0.63	0.10
TPV (ms)	599.32 ± 265.85	438.22 ± 223.70	[48.54, 273.64]	**0.01**	0.64
EI	13.29 ± 4.78	13.55 ± 5.52	[−2.62, 2.09]	0.82	0.05
MPP (W)	282.02 ± 60.60	266.63 ± 59.64	[−8.65, 39.43]	0.20	0.28
PP (W)	456.02 ± 142.37	410.35 ± 86.52	[−15.66, 107.00]	0.14	0.33
BD (cm)	40.74 ± 3.95	39.77 ± 4.58	[−0.50, 2.44]	0.18	0.29
SET 3					
MPV (m·s^−1^)	0.45 ± 0.11	0.43 ± 0.07	[−0.03, 0.06]	0.44	0.17
PV (m·s^−1^)	0.70 ± 0.20	0.65 ± 0.12	[−0.04, 0.13]	0.28	0.24
VL (%)	30.72 ± 11.74	26.02 ± 6.96	[0.76, 8.64]	**0.02**	0.53
TPV (ms)	498.36 ± 259.04	532.73 ± 258.87	[−143.16, 74.43]	0.52	0.14
EI	13.76 ± 5.83	11.29 ± 3.50	[0.63, 4.31]	**0.01**	0.60
MPP (W)	274.94 ± 64.94	266.46 ± 59.35	[−15.42, 32.37]	0.47	0.16
PP (W)	460.10 ± 146.43	411.45 ± 90.56	[−10.35, 107.64]	0.10	0.37
BD (m)	40.51 ± 4.50	39.09 ± 3.79	[−0.02, 2.85]	0.06	0.44

Significant differences are marked in bold. Data are presented as means ± SD unless otherwise stated. Bold values denote significant differences. W + SQ + BP: Warm-Up + Squat + Bench Press; W + SQ + RW + BP: Warm-Up + Squat + Specific Re-Warm-Up + Bench Press; BD: bar displacement; CI: confidence interval; ES: Cohen’s *d* effect size; MPV: mean propulsive velocity; PV: peak velocity; VL: velocity loss; TPV: time to achieve peak velocity; EI: effort index; MPP: mean propulsive power; PP: peak power.

## Data Availability

Data will be made available upon reasonable request.

## References

[1] Ayala F., Moreno-Pérez V., Vera-Garcia F.J., Moya M., Sanz-Rivas D., Fernandez-Fernandez J. (2016). Acute and Time-Course Effects of Traditional and Dynamic Warm-Up Routines in Young Elite Junior Tennis Players. PLoS ONE.

[2] Barnes M.J., Petterson A., Cochrane D.J. (2017). Effects of Different Warm-up Modalities on Power Output during the High Pull. J. Sports Sci..

[3] Neiva H.P., Marques M.C., Fernandes R.J., Viana J.L., Barbosa T.M., Marinho D.A. (2014). Does Warm-Up Have a Beneficial Effect on 100-m Freestyle?. Int. J. Sports Physiol. Perform..

[4] Zois J., Bishop D., Aughey R. (2015). High-Intensity Warm-Ups: Effects During Subsequent Intermittent Exercise. Int. J. Sports Physiol. Perform..

[5] Fradkin A.J., Zazryn T.R., Smoliga J.M. (2010). Effects of Warming-up on Physical Performance: A Systematic Review with Meta-Analysis. J. Strength Cond. Res..

[6] McGowan C.J., Pyne D.B., Thompson K.G., Rattray B. (2015). Warm-Up Strategies for Sport and Exercise: Mechanisms and Applications. Sports Med..

[7] Andrade D.C., Henriquez-Olguin C., Beltran A.R., Ramirez M.A., Labarca C., Cornejo M., Alvarez C., Ramirez-Campillo R. (2015). Effects of General, Specific and Combined Warm-up on Explosive Muscular Performance. Biol. Sport.

[8] Van Den Tillaar R., Vatten T., Von Heimburg E. (2017). Effects of Short or Long Warm-up on Intermediate Running Performance. J. Strength Cond. Res..

[9] Chiba I., Samukawa M., Takizawa K., Nishikawa Y., Ishida T., Kasahara S., Yamanaka M., Tohyama H. (2022). Warm-Up Intensity and Time-Course Effects on Jump Height under Cold Conditions. Int. J. Environ. Res. Public Health.

[10] Tsurubami R., Oba K., Samukawa M., Takizawa K., Chiba I., Yamanaka M., Tohyama H. (2020). Warm-Up Intensity and Time Course Effects on Jump Performance. J. Sports Sci. Med..

[11] Alves M.D.D.J., Knechtle B., Silva D.D.S., Fernandes M.S.D.S., Gomes J.H., Thuany M., Aidar F.J., Weiss K., De Souza R.F. (2023). Effects of High-Intensity Warm-Up on 5000-Meter Performance Time in Trained Long-Distance Runners. J. Sports Sci. Med..

[12] Alves R.R., Viana R.B., Silva M.H., Guimarães T.C., Vieira C.A., Santos D.D.A.T., Gentil P.R.V. (2021). Postactivation Potentiation Improves Performance in a Resistance Training Session in Trained Men. J. Strength Cond. Res..

[13] Garbisu-Hualde A., Santos-Concejero J. (2021). Post-Activation Potentiation in Strength Training: A Systematic Review of the Scientific Literature. J. Hum. Kinet..

[14] Batista M.A., Roschel H., Barroso R., Ugrinowitsch C., Tricoli V. (2011). Influence of Strength Training Background on Postactivation Potentiation Response. J. Strength Cond. Res..

[15] Wyland T.P., Van Dorin J.D., Reyes G.F.C. (2015). Postactivation Potentation Effects From Accommodating Resistance Combined With Heavy Back Squats on Short Sprint Performance. J. Strength Cond. Res..

[16] Chaouachi A., Castagna C., Chtara M., Brughelli M., Turki O., Galy O., Chamari K., Behm D.G. (2010). Effect of Warm-Ups Involving Static or Dynamic Stretching on Agility, Sprinting, and Jumping Performance in Trained Individuals. J. Strength Cond. Res..

[17] Kilduff L.P., Owen N., Bevan H., Bennett M., Kingsley M.I.C., Cunningham D. (2008). Influence of Recovery Time on Post-Activation Potentiation in Professional Rugby Players. J. Sports Sci..

[18] Wilson J.M., Duncan N.M., Marin P.J., Brown L.E., Loenneke J.P., Wilson S.M.C., Jo E., Lowery R.P., Ugrinowitsch C. (2013). Meta-Analysis of Postactivation Potentiation and Power: Effects of Conditioning Activity, Volume, Gender, Rest Periods, and Training Status. J. Strength Cond. Res..

[19] Abad C.C., Prado M.L., Ugrinowitsch C., Tricoli V., Barroso R. (2011). Combination of General and Specific Warm-Ups Improves Leg-Press One Repetition Maximum Compared with Specific Warm-Up in Trained Individuals. J. Strength Cond. Res..

[20] Neves P.P., Alves A.R., Marinho D.A., Ferraz R., Garrido N., Marques M.C., Neiva H.P. (2024). The Impact of General and/or Specific Warm-up on Power and Velocity during Squat and Bench-Press Training. Retos.

[21] Ribeiro B., Pereira A., Neves P.P., Sousa A.C., Ferraz R., Marques M.C., Marinho D.A., Neiva H.P. (2020). The Role of Specific Warm-up during Bench Press and Squat Exercises: A Novel Approach. Int. J. Environ. Res. Public Health.

[22] Ribeiro B., Pereira A., Alves A.R., Neves P.P., Marques M.C., Marinho D.A., Neiva H.P. (2021). Specific Warm-up Enhances Movement Velocity during Bench Press and Squat Resistance Training. J. Men’s Health.

[23] González-Badillo J.J., Sánchez-Medina L., Ribas-Serna J., Rodríguez-Rosell D. (2022). Toward a New Paradigm in Resistance Training by Means of Velocity Monitoring: A Critical and Challenging Narrative. Sports Med.—Open.

[24] Morán-Navarro R., Martínez-Cava A., Sánchez-Medina L., Mora-Rodríguez R., González-Badillo J.J., Pallarés J.G. (2019). Movement Velocity as a Measure of Level of Effort During Resistance Exercise. J. Strength Cond. Res..

[25] González-Badillo J.J., Sánchez-Medina L. (2010). Movement Velocity as a Measure of Loading Intensity in Resistance Training. Int. J. Sports Med..

[26] Ribeiro A.S., Romanzini M., Schoenfeld B.J., Souza M.F., Avelar A., Cyrino E.S. (2014). Effect of Different Warm-up Procedures on the Performance of Resistance Training Exercises. Percept. Mot. Skills.

[27] Ribeiro B., Pereira A., Neves P., Marinho D., Marques M., Neiva H.P. (2021). The Effect of Warm-up in Resistance Training and Strength Performance: A Systematic Review. Motricidade.

[28] Simão R., De Salles B.F., Figueiredo T., Dias I., Willardson J.M. (2012). Exercise Order in Resistance Training. Sports Med..

[29] Kapnia A.Κ., Dallas C.N., Gerodimos V., Flouris A.D. (2023). Impact of Warm-Up on Muscle Temperature and Athletic Performance. Res. Q. Exerc. Sport.

[30] Faul F., Erdfelder E., Lang A.-G., Buchner A. (2007). G*Power 3: A Flexible Statistical Power Analysis Program for the Social, Behavioral, and Biomedical Sciences. Behav. Res. Methods.

[31] Rodríguez-Rosell D., Yanez-Garcia J.M., Torres-Torrelo J., Mora-Custodio R., Marques M.C., González-Badillo J.J. (2018). Effort Index as a Novel Variable for Monitoring the Level of Effort During Resistance Exercises. J. Strength Cond. Res..

[32] Kompf J., Arandjelović O. (2017). The Sticking Point in the Bench Press, the Squat, and the Deadlift: Similarities and Differences, and Their Significance for Research and Practice. Sports Med..

[33] Benavides-Ubric A., Díez-Fernández D.M., Rodríguez-Pérez M.A., Ortega-Becerra M., Pareja-Blanco F. (2020). Analysis of the Load-Velocity Relationship in Deadlift Exercise. J. Sports Sci. Med..

[34] Kilgallon J., Cushion E., Joffe S., Tallent J. (2022). Reliability and Validity of Velocity Measures and Regression Methods to Predict Maximal Strength Ability in the Back-Squat Using a Novel Linear Position Transducer. Proc. Inst. Mech. Eng. Part P J. Sports Eng. Technol..

[35] Pallarés J.G., Sánchez-Medina L., Pérez C.E., De La Cruz-Sánchez E., Mora-Rodriguez R. (2014). Imposing a Pause between the Eccentric and Concentric Phases Increases the Reliability of Isoinertial Strength Assessments. J. Sports Sci..

[36] Sánchez-Medina L., González-Badillo J.J., Perez C.E., Pallares J.G. (2014). Velocity- and Power-Load Relationships of the Bench Pull vs. Bench Press Exercises. Int. J. Sports Med..

[37] Sánchez-Medina L., González-Badillo J. (2011). Velocity Loss as an Indicator of Neuromuscular Fatigue during Resistance Training. Med. Sci. Sports Exerc..

[38] Sánchez-Medina L., Pallarés J.G., Pérez C.E., Morán-Navarro R., González-Badillo J.J. (2017). Estimation of Relative Load From Bar Velocity in the Full Back Squat Exercise. Sports Med. Int. Open.

[39] Marques D.L., Neiva H.P., Faíl L.B., Gil M.H., Marques M.C. (2019). Acute Effects of Low and High-Volume Resistance Training on Hemodynamic, Metabolic and Neuromuscular Parameters in Older Adults. Exp. Gerontol..

[40] Resende M.D.A., Vasconcelos Resende R.B., Reis G.C., Barros L.D.O., Bezerra M.R.S., Matos D.G.D., Marçal A.C., Almeida-Neto P.F.D., Cabral B.G.D.A.T., Neiva H.P. (2020). The Influence of Warm-Up on Body Temperature and Strength Performance in Brazilian National-Level Paralympic Powerlifting Athletes. Medicina.

[41] Borg G.A. (1982). Psychophysical Bases of Perceived Exertion. Med. Sci. Sports Exerc..

[42] Hopkins W.G., Marshall S.W., Batterham A.M., Hanin J. (2009). Progressive Statistics for Studies in Sports Medicine and Exercise Science. Med. Sci. Sports Exerc..

[43] Contreras B., Vigotsky A.D., Schoenfeld B.J., Beardsley C., Cronin J. (2016). A Comparison of Gluteus Maximus, Biceps Femoris, and Vastus Lateralis Electromyography Amplitude in the Parallel, Full, and Front Squat Variations in Resistance-Trained Females. J. Appl. Biomech..

[44] Sanchis-Moysi J., Idoate F., Olmedillas H., Guadalupe-Grau A., Alayón S., Carreras A., Dorado C., Calbet J.A.L. (2010). The Upper Extremity of the Professional Tennis Player: Muscle Volumes, Fiber-type Distribution and Muscle Strength. Scand. Med. Sci. Sports.

[45] Bishop D. (2003). Warm Up I: Potential Mechanisms and the Effects of Passive Warm Up on Exercise Performance. Sports Med..

[46] Bishop D. (2003). Warm Up II: Performance Changes Following Active Warm Up and How to Structure the Warm Up. Sports Med..

[47] Ratamess N.A., Alvar B.A., Evetoch T.K., Housh T.J., Kibler W.B., Kraemer W.J., Triplett N.T. (2009). Progression Models in Resistance Training for Healthy Adults. Med. Sci. Sports Exerc..

[48] McMahon S., Jenkins D. (2002). Factors Affecting the Rate of Phosphocreatine Resynthesis Following Intense Exercise. Sports Med..

